# A comparison of nanoparticullate CpG immunotherapy with and without allergens in spontaneously equine asthma‐affected horses, an animal model

**DOI:** 10.1002/iid3.198

**Published:** 2017-11-01

**Authors:** John Klier, Sabine Geis, Jeanette Steuer, Katharina Geh, Sven Reese, Sebastian Fuchs, Ralf S. Mueller, Gerhard Winter, Heidrun Gehlen

**Affiliations:** ^1^ Centre for Clinical Veterinary Medicine Equine Clinic, Ludwig‐Maximilians‐University Munich Germany; ^2^ Department of Pharmacy Pharmaceutical Technology and Biopharmaceutics, Ludwig‐Maximilians‐University Munich Germany; ^3^ Department of Veterinary Science, Institute of Anatomy, Histology and Embryology Ludwig‐Maximilians‐University Munich Germany; ^4^ Centre for Clinical Veterinary Medicine, Small Animal Medicine Clinic Ludwig‐Maximilians‐University Munich Germany; ^5^ Department of Veterinary Medicine, Equine Clinic, Surgery and Radiology Free University of Berlin Berlin Germany

**Keywords:** allergic asthma, extrinsic asthma, heaves, inhalation, neutrophilic asthma

## Abstract

**Introduction:**

New therapeutic strategies to modulate the immune response of human and equine allergic asthma are still under extensive investigation. Immunomodulating agents stimulating T‐regulatory cells offer new treatment options beyond conventional symptomatic treatment or specific immunotherapy for human and equine allergic airway diseases, with the goal of a homoeostatic T‐helper cell balance. The aim of this study was to evaluate the effects of a nebulized gelatin nanoparticle‐CpG formulation (CpG‐GNP) with and without specific allergens for the treatment of spontaneous allergic equine asthma as a model for human asthma.

**Methods:**

Twenty equine asthma‐affected horses were treated either with CpG‐GNP alone or CpG‐GNP with allergens. Two specific allergens were selected for each horse based on history and an in‐vitro test. Each horse received seven administrations of the respective nebulized composition and was examined before treatment, immediately after and 6 weeks after the treatment course.

**Results:**

Clinical parameters such as breathing rate, indirect interpleural measurement, arterial blood gases, amount of tracheal mucus and percentage of neutrophils and cytokines in tracheal washes and serum samples were evaluated. Treatment with CpG‐GNP alone as well as in combinations with relevant allergens resulted in clinical improvement of nasal discharge, breathing rate, amount of secretion and viscosity, neutrophil percentage and partial oxygen pressure directly after and 6 weeks after treatment. There were no significant differences between the two treatments in clinical parameters or local cytokine profiles in the tracheal wash fluid (IL‐10, IFN‐g, and IL‐17). IL‐4 concentrations decreased significantly in both groups.

**Conclusion:**

Nonspecific CpG‐GNP‐based immunotherapy shows potential as a treatment for equine and possibly also human allergic asthma.

## Introduction

Allergic airway diseases have increased over the last few decades 1. Equine asthma (Recurrent airway obstruction, RAO, heaves) is among the most frequent equine allergic airway diseases in the Northern Hemisphere 2, 3. The pathophysiology of equine asthma resembles that of human allergic neutrophilic asthma and could serve as spontaneous animal model 3, 4. Pathophysiology includes cholinergic bronchospasm, hypercrinia and dyscrinia, edema, airway neutrophilia, and airway remodeling, resulting in nasal discharge, coughing, and poor performance 2, 3, 4. Potential allergic triggers are organic and inorganic substances from hay dust and straw 2, 3. The predominant stabling of horses in box stalls in our latitudes increases the exposure to these triggers. In addition, a genetic predisposition has been reported 5. The exact nature of those environmental trigger factors for equine asthma, such as pollens, mold spores or storage mites is still not known. The avoidance of allergens (hay dust, mold spores from straw) currently constitutes the most important therapeutic measure, besides symptomatic therapy with glucocorticoids and bronchodilators. As absolute avoidance of allergens is difficult to impossible and treatment with long‐term medications may be associated with adverse effects, there is a great need for new therapeutic concepts.

Immunotherapy provides a promising approach by stimulating regulatory T cells (Treg), with the goal of reestablishing the homeostasis of the disrupted T‐helper cell balance. Nanoparticle‐bound CpG‐ODNs (Cytosine‐phosphate‐guanosine oligodeoxynucleotides, CpG‐GNP) were reported as an effective immunomodulatory treatment for human and equine asthma 6, 7, 8. CpG is recognized by a type of Toll‐like receptor (TLR9) within the endosomal compartments. This leads to a stimulation of subclasses of T‐lymphocytes (Th1, Treg) and cytokine synthesis (IFN‐γ, IL‐10). Macrophages, epithelial cells and neutrophilic granulocytes expressing TLR9 have already been detected in equine lungs 9. The stimulation of the Th1 response in turn causes a downregulation of the allergen‐specific Th2‐type response (Th2/Th1 shift). Furthermore, regulatory cytokines such as the anti‐inflammatory IL‐10 are upregulated 10.

Gelatin nanoparticles (GNP) are immunologically inert and biodegradable 11. They are inserted as carriers for the oligodeoxynucleotides and prevent the premature degradation of DNA through ubiquitous DNAses. Furthermore, the absorption through endocytosis and thus the effect of the CpG is increased in the target cells 11. This delivery system is efficacious in‐vitro in BAL cells and in‐vivo in the horse 7, 8, 12.

The goal of the present study was to determine whether monotherapy with CpG‐GNP alone is as efficacious for equine asthma as CpG‐GNP combined with specific allergens in a protocol using inhalation therapy over a longer time period compared to previous studies 7, 8. For this purpose, clinical, endoscopic, cytological, and immunological parameters were determined both locally in the lungs and systemically.

## Methods

### Study design and patients

In this prospective randomized exploratory field study, twenty horses (mean age 17 ± 5 years; 15 geldings, 4 mares, 2 stallions; mean weight determined via weight tape 498 kg ± 111 kg) with a chronic history (mean 7 ± 5 years, range 1–19 years; mean disease onset 10 ± 4 years) of equine asthma were admitted to the study. The study was approved by the regional legal agency for animal experiments of the Government of Upper Bavaria, Germany (No. 55.2‐1‐54‐2531‐31‐10). All horse owners signed an informed consent form. The study has been performed in compliance with the guidelines for animal studies (ARRIVE) and clinical trials (CONSORT). The subjects were randomly selected for one of the two treatment groups via coin toss.

### Inclusion criteria

The inclusion criteria were a raised breathing rate at rest (>16/min), an increased abdominal breathing pattern, pathological findings of pulmonary auscultation, increased levels of neutrophilic granulocytes in the respiratory tract (>25%), reduced arterial blood gas levels at rest (partial oxygen pressure <90 mmHg), increased interpleural pressure (>15 cmH_2_O), inducible clinical signs upon contact with dusty hay and straw, and resolution of clinical signs, when the horse was kept outside 2. All of the horses were left in their customary stabling and feed conditions during the entire study, and were examined in their home stables. During the study (performed between June and November), no changes occurred in the keeping or management of the horses. In addition, none of the horses received any additional medications for at least 8th weeks before the start and throughout the duration of the study.

### Allergy testing

A functional in‐vitro test from whole blood (FIT) 13 was carried out on all horses by the Institute for Immunology at the University of Veterinary Medicine Hannover. The blood was sent overnight by express delivery.

### Intervention

One group (*n* = 11, allergen group) received CpG‐GNP in combination with two individually selected allergens (Table 1). The second group (*n* = 9, CpG only group) received only CpG‐GNP^1^ ^6^. The lower number of patients in the pure CpG group was because of drop out for reasons not correlated to the study (e.g., lameness with medical treatment). Allergens were received from Artu Biologicals Europe B.V. (Lelystad, the Netherlands) and diluted 1:1000 with phosphate buffered salt solution (PBS; Dulbeco, Biochrom AG, Berlin, Germany) to avoid acute signs of bronchospasm. The allergens for inhalation were selected based on history (local geographical distribution of the allergens and seasonality of clinical signs) and allergy test results. The amount of allergen extract was increased gradually from 0.6 ml (0.3 ml per allergen) for two inhalations, to 0.4 ml per allergen for two inhalations, followed by 0.5 ml per allergen for two inhalations and finally 0.6 ml per allergen for the final seventh inhalation.

**Table 1:** List of individual chosen allergens for every horse in the allergen & CpG‐GNP treatment group (*n* = 11 equine asthma‐affected horses)

	Allergen I	Allergen II
Horse 1	*Acarus siro* (Flour mite)	*Alopecurus pratensis* (Meadow foxtail)
Horse 2	*Brassica napus* (Rapeseed)	*Lepidoglyphus destructor* (Storage mite)
Horse 3	*Aspergillus fumigatus* (Mold)	*Acarus siro* (Flour mite)
Horse 4	*Acarus siro* (Flour mite)	*Dermatophagoides pteronyssinus* (House dust mite)
Horse 5	*Acarus siro* (Flour mite)	*Dermatophagoides peronyssinus* (House dust mite)
Horse 6	*Aspergillus fumigatus* (Mold)	*Acarus siro* (Flour mite)
Horse 7	*Lepidoglyphus destructor* (Hay mite)	*Dermatophagoides pteronyssinus* (House dust mite)
Horse 8	*Aspergillus fumigatus* (Mold)	*Dermatophagoides pteronyssinus* (House dust mite)
Horse 9	*Aspergillus fumigatus* (Mold)	*Brassica napus* (Rapeseed)
Horse 10	*Brassica napus* (Rapeseed)	Non

Horse number 10 had only one positive allergen reaction in the allergy test.

Nanoparticles were produced and loaded with CpG‐ODN 2216 (Biomers GmbH, Ulm, Germany) as previously described 7, 8. Each inhalation utilized 187.5 µg CpG‐ODN, bound to 3.75 mg GNP and dispersed in 2.5 ml highly purified water (HPW), with a final concentration of 1.5 mg/ml GNP and 0.075 mg/ml CpG‐ODN.

The individual dosage of CpG‐GNP remained the same in both groups. The inhalation regimen was conducted with Equine Haler®™,^2^ and Aeroneb® Go micropump nebulizer^3^ as previously described 7, 8.

The seven inhalations in each horse were administered every other day. The interventions were always carried out by two individuals (SG, JS).

### Clinical and laboratory evaluations

The first evaluation of the horses occurred before the treatment (I) and included clinical evaluation, bronchoscopy, and evaluation of pulmonary function parameters, as well as cytological, immunological, and laboratory‐chemical parameters. The second evaluation (II) occurred after the completion of the seventh inhalation 2 weeks later. The third evaluation (III) occurred 6 weeks after the last treatment, in order to determine any long‐term effect.

The clinical examination was conducted as previously reported 7, 8 and in accordance with established and standardized scoring systems 2, 14, 15, where available. The variables examined included the measurement of the following: breathing rate at rest, breathing type (relative units [RU] in the range of 0–4; where 0 = no or little movement in the ventral region of the flank, and 4 = obvious abdominal lift, heave line and nostril flaring) 14, nasal discharge (RU in the range of 0–3; 0 = none, 1 = mild, 2 = moderate, and 3 = severe discharge), auscultation of lungs and trachea (in the range of RU 0–4; 0 = physiological, 1 = mild, 2 = moderate, 3 = severe, 4 = wheezes/rhonchi) 2, 7, 8, and arterial blood gas parameters 15. The partial pressures of oxygen and carbon dioxide (PaO_2_, PaCO_2_) were determined. The physiological values were set at 100 mmHg ± 5 mmHg for PaO_2_ and 40 mmHg ± 5 mmHg for PaCO_2_ 15. The arterial blood gas values were measured via the IRMA® blood analysis system.^4^ The alveolar‐arterial oxygen gradient (AaDO2) was calculated according to the current atmospheric pressure and the measured blood gas values [AaDO2 = (atmospheric pressure −47 mmHg) × 0.2095‐ PaCO_2_ ‐ PaO_2_] 15. In addition, an endoscopic examination and a quantification of secretion (grade 0–5, 0 = none, clean, singular tracheobronchial secretion [TBS] to 5 = extreme, profuse amounts) 16 and viscosity (grade 1 = fluid to 5 = viscous) 16 of the TBS was performed. Cytological examinations of the TBS samples were performed to determine the percentage of neutrophils in the total cell count after staining with Diff‐Quick solution.^5^ Indirect measurement of the maximum interpleural pressure differences (D Ppl max) was performed by a Venti‐Graph via esophageal probe 17.^6^

In order to monitor the tolerability of the treatment, the patients were continuously observed throughout the duration of the experiment, with written record of any nasal discharge (consistency and quantity), coughing (at rest and during exercise), signs of bronchospasm (respiratory sounds such as wheezing and whistling, flared nostrils, increased abdominal breathing type), increased breathing rate at rest, redness or swelling visible by endoscopy, follicular hyperplasia, or reduced arterial blood gas values. In addition, the internal body temperature was measured regularly, and, within the scope of the three examinations, a white blood cell count, a differential blood count, and a fibrinogen test were evaluated for signs of adverse systemic effects.

Tracheal wash was performed with 20 ml of sterile saline. Venous blood samples and tracheal wash fluid were centrifuged and the supernatant was immediately frozen with liquid nitrogen and stored at −80°C until the samples were evaluated. The concentration of the cytokines IL‐4, IL‐10, and IFN‐gamma were measured in both serum and tracheal wash fluid, IL‐17 was only evaluated in the tracheal wash fluid. The equine sandwich ELISAs were carried out as described before 18 in compliance with the manufacturer's protocol.^7^ Samples above the detection limit were diluted with sterile sodium chloride solution and the ELISA was repeated. The dilution factor was used to calculate the exact concentration of the sample. Sodium chloride was also used as negative control and results of photometric extinction were subtracted from the detected values of the samples. In some cases this led to values below the detection limit (after subtraction of the negative control).

### Statistical analysis

Sample size calculation was performed (one‐tailed, Cohen‘s d >1 for relevant clinical effect with a power of >0.8 and α *P* = 0.05) with 12 subjects per group. For non‐parametric data, the Wilcoxon matched‐pairs signed‐rank test was used for paired values (comparison within the groups) and the Mann‐Whitney test was used for unpaired data (comparison between the groups). For multiple tests a Kruskal–Wallis test with post hoc Dunn test was performed for clinical, endoscopic, cytological, and biochemical parameters (excluding cytokine values). Only non‐parametric tests were used because of low numbers of individuals per group. All statistical analyses were performed using the GraphPad Prism 7 software.^9^ Results with a calculated value of *P* < 0.05 were considered as statistically significant (displayed as *; *P* < 0.01 as **; *P* < 0.001 as ***) and were reported as mean ± SD for normally distributed data (clinical parameter) or median with interquartile range (IQR) for not normally distributed data (cytokine values) according to Shapiro‐Wilk test. The 95% confidence intervals (CI) were calculated and presented in brackets. Effect size (Cohen's d) was assessed for each piece of data to quantify the effect (mild, *d* = 0.2–0.3; moderate, *d* = 0.5; large, *d* > 0.8). The calculation of the effect size was performed using the Effect Size Calculator^8^ ^19^.

## Results

### Allergy testing

**Figure 1:**
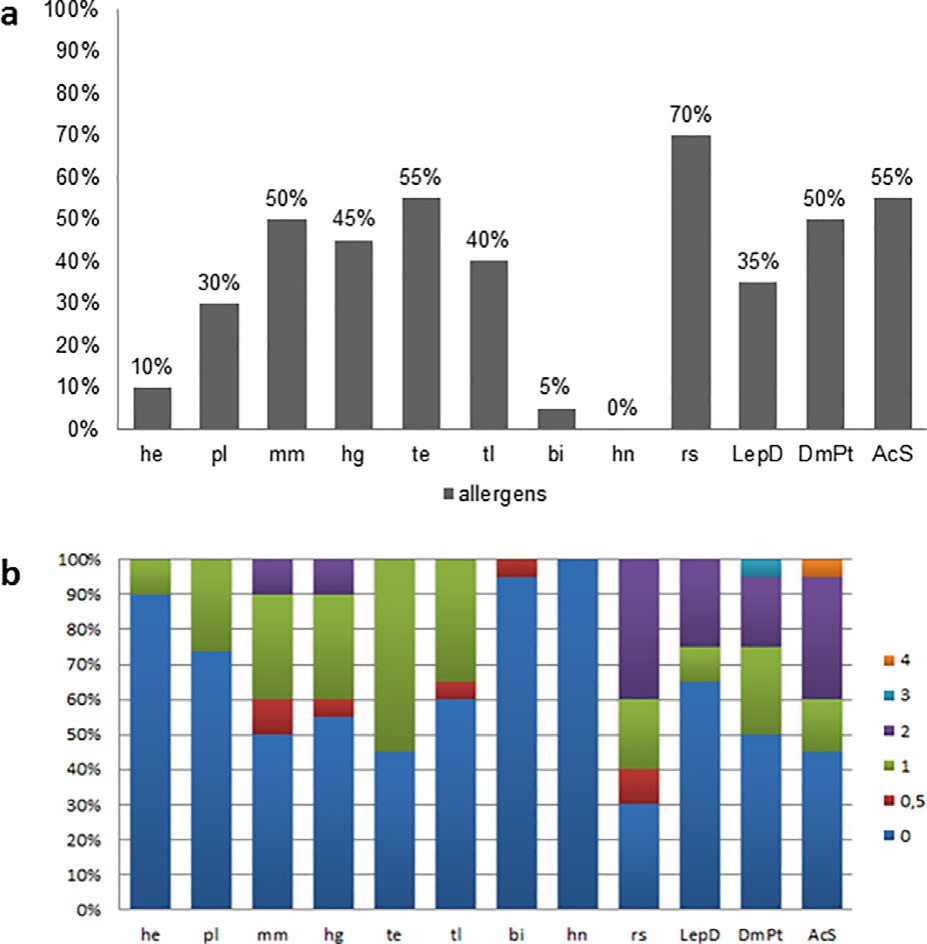
Proportionate results from allergen tests concerning individual allergens (a, b). Numbers and colors illustrate individual reactivity of type I immunoreaction and horses: 0 = no reaction, 1 = clearly positive to 4 = high grade positive (He, herbage; pl, pollen late; mm, mold mix; hg, herbage grain; te, tree early; tl, tree late; bi, birch; hn, hazelnut; rs, rape; LepD, Lep. Destr.; DmPt, Derm. Pteron.; AcS, Acarus siro).

The most frequently positive result (70%) was to rapeseed (rs; Brassica napus) (Fig. 1a). Half of the equine asthma horses reacted positively to mold mix (mm), tree early (te) and mites such as Dermatophagoides pteronyssinus (DermPt) and Acarus siro (AcS) (Fig. 1a). A sensitivity of the equine asthma horses was also frequently observed to herbage grain (hg; 45%), tree late (tl, 40%) and Lepidoglyphus destructor (LepD; 35%). Birch (bi) and herbage (He) were very infrequently positive (Fig. 1a). No horse reacted to hazelnut (hz) (Fig. 1a). The most severe reaction (grade 4) was observed with AcS (Fig. 1b), followed by other mites such as DermPt, LepD, rs, mm, and hg (Fig. 1b).

### Adverse effects

Throughout the entire study, no signs of local or systemic adverse effects were observed as a result of the inhalation treatment. No bronchospasm, endoscopically visible local hyperemia, swelling, follicular hyperplasia, or reduced arterial blood gas values were observed. Furthermore, no increase in internal body temperature, number of leukocytes, differential blood count, or fibrinogen was measured.

### Comparison between the groups

**Figure 2:**
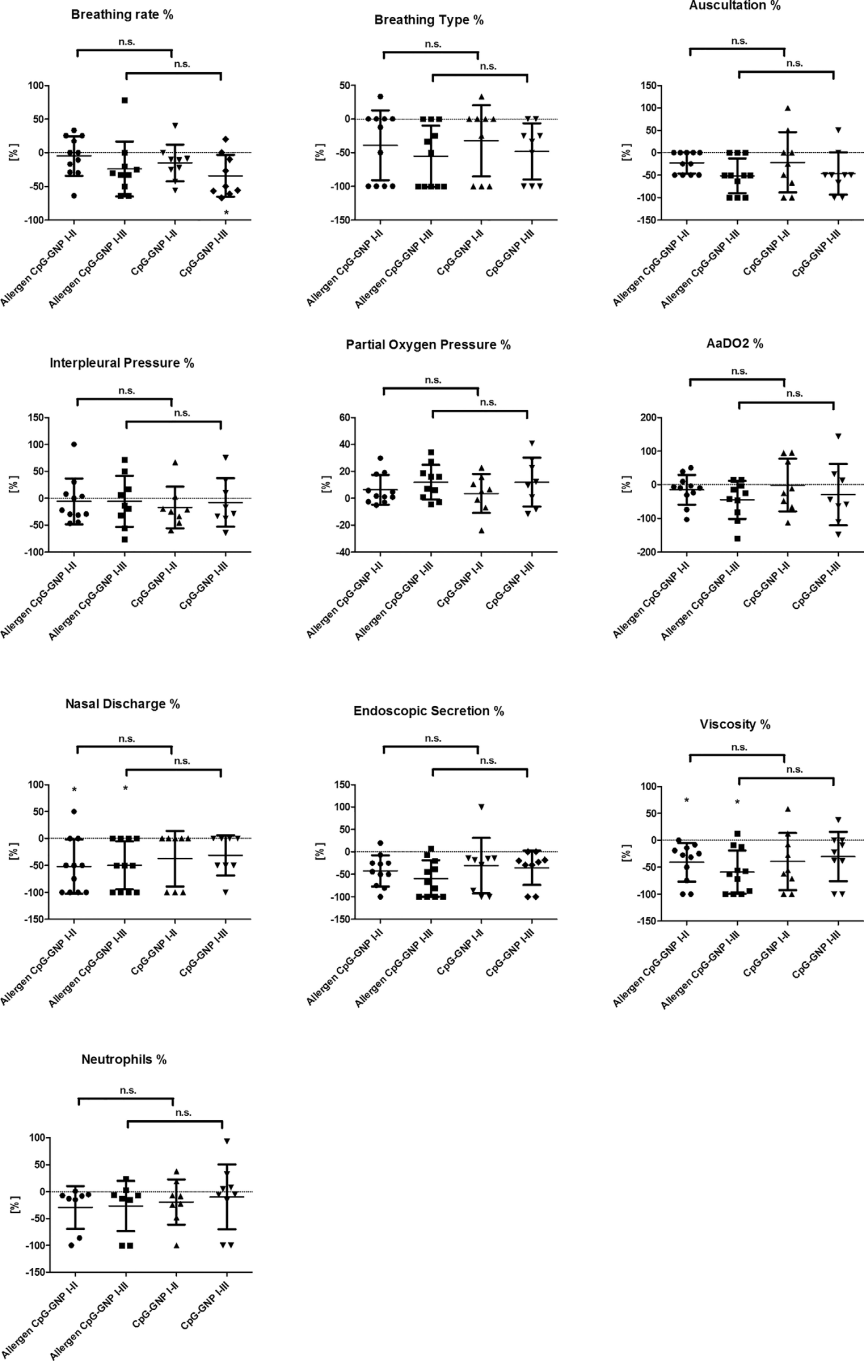
Relative decrease or increase [%] of breathing rate and pattern (calculated value of *P* < 0.05 displayed as *; *P* < 0.01 as **; *P* < 0.001 as ***), lung auscultation, interpleural pressure measurement, oxygen partial pressure, AaDO2, percentage of neutrophils in TBS, nasal discharge, endoscopic quantification of secretion and viscosity depicted in percent between first and second (I–II) and first and third (I–III) examinations and in comparison between combined allergen and CpG‐GNP treatment group (*n* = 11 equine asthma‐affected horses) and pure CpG‐GNP (*n* = 9 equine asthma‐affected horses) group (mean ± SD).

Comparing the two groups (CpG only vs. allergen group), there was no significant difference at any time point of examination concerning all measured parameters including breathing rate, breathing type, lung auscultation, interpleural pressure, alveolar‐arterial gradient (AaDO_2_), oxygen partial pressure (PaO_2_), percentage of neutrophils, nasal discharge, quantification of secretion (mucus), and viscosity of secretion (Fig. 2).

### Breathing rate

In the allergen group, the breathing rate showed no clinically relevant decrease after seven inhalations (Fig. 2, Tables 2 and 3). In the subsequent 6‐week interval without treatment, the breathing rate markedly improved (Fig. 2, Tables 2 and 3) and showed a mean reduction of the breathing rate of 24% in comparison to the initial value. In the CpG only group a mild to moderate clinical improvement (Fig. 2, Tables 2 and 3) of the breathing rate of 15% was already observed upon completion of the seven inhalations. This effect strongly increased to mean reduction of 34% in the subsequent 6‐week period without treatment and was statistically significant (Fig. 2, Tables 2 and 3) in comparison to the initial value.

**Table 2:** Results of breathing rate [1/min], breathing type [RU 0–4], lung auscultation [RU 0–4], interpleural pressure measurement [cmH2O], oxygen partial pressure (PO2) [mmHg], AaDO2 [mmHg], neutrophil percentage of TBS [%], nasal discharge [RU 0–3], endoscopic quantification of secretion (mucus) [RU 0–5] and viscosity [RU 0–5] depicted before (I), after 7 inhalations (II), and 6 weeks post‐treatment (III) with allergen & CpG treatment (*n* = 11 equine asthma‐affected horses) versus pure CpG treatment (*n* = 9 equine asthma‐affected horses) (RU = relative units, scoring system; mean ±SD)

	Breathing rate [1/min]			Breathing type [RU 0–4]			Auscultation [RU 0–4]			Interpleural pressure [cmH_2_O]		
	I	II	III	I	II	III	I	II	III	I	II	III
Allergen+CpG (*n* = 11)	22.9 ± 7.1	21.1 ± 7.9	16.4 ± 8.0	2.5 ± 1.2	1.6 ± 1.7	1.1 ± 1.2	2.1 ± 1.3	1.7 ± 1.2	1.0 ± 0.7	28.5 ± 25.1	26.0 ± 24.2	22.8 ± 22.4
CpG (*n* = 9)	30.6 ± 10.2	25.3 ± 8.5	19.6 ± 11.3	2.9 ± 1.0	1.9 ± 1.6	1.6 ± 1.2	2.4 ± 1.1	1.7 ± 1.4	1.1 ± 1.0	23.8 ± 22.9	21.0 ± 21.8	16.9 ± 16.8

	AaDO2 [mmHg]			PO_2_ [mmHg]			Neutrophil percentage [%]		
	I	II	III	I	II	III	I	II	III
Allergen+ CpG (*n* = 11)	23.1 ± 9.4	20.6 ± 15.3	13.0 ± 14.0	80.2 ± 11.8	85.3 ± 15.2	91.1 ± 15.6	68.2 ± 36.5	57.9 ± 34.3	51.8 ± 39.1
CpG (*n* = 9)	18.6 ± 9.0	18.6 ± 13.7	12.3 ± 12.2	86.3 ± 12.7	89.1 ± 13.1	95.4 ± 11.9	63.1 ± 34.6	63.8 ± 32.0	67.5 ± 39.0

	Nasal discharge [RU 0–3]			Quantification of secretion [RU 0–5]			Viscosity [RU 0–5]		
	I	II	III	I	II	III	I	II	III
Allergen+ CpG (*n* = 11)	1.5 ± 0.5	0.6 ± 0.6	0.7 ± 0.5	3.0 ± 1.1	1.8 ± 1.3	1.6 ± 1.5	3.3 ± 0.7	2.0 ± 1.2	1.6 ± 1.5
CpG (*n* = 9)	1.3 ± 0.7	0.8 ± 0.7	0.8 ± 0.6	3.1 ± 1.1	2.3 ± 1.7	2.2 ± 1.4	3.0 ± 1.3	1.8 ± 1.5	2.2 ± 1.3

**Table 3:** Cohen's d, confidence interval (CI) and *P*‐value calculated from breathing rate, breathing type, lung auscultation, interpleural pressure measurement, oxygen partial pressure (PO2), AaDO2, neutrophil percentage ofTBS, nasal discharge, endoscopic quantification of secretion (mucus) and viscosity from proportional values [%] between first and second (I–II) or first and third (I–III) examination from allergen + CpG group (allergen) versus CpG only group (CpG)

	Breathing rate	Breathing type	Auscultation	Interpleural pressure
I‐II Allergen versus CpG [%]	*d* = −0.37 CI:−1.24 to 0.53 *P* = 0.4694	*d* = 0.13 CI:−0.75 to 1.01 *P* = 0.7813	*d* = 0.04 CI:−0.85 to 0.91 *P* = 0.8141	*d* = −0.05 CI:−0.93 to 0.84 *P* = 0.5915
I‐III Allergen versus CpG [%]	*d* = −0.27 CI:−1.17 to 0.64 *P* = 0.7128	*d* = 0.39 CI:−054 to 1.28 *P* = 0.7785	*d* = 0.01 CI:−0.89 to 0.91 *P* = 0.9683	*d* = −0.05 CI:−1.0 to 0.91 *P* = 0.8884

	AaDO_2_	PO_2_	Neutrophil percentage
I‐II Allergen versus CpG [%]	*d* = 0.23 CI:−0.69 to 1.13 *P* = 0.9671	*d* = 0.22 CI:−0.7 to 1.12 *P* = 0.9671	*d* = 0.25 CI:−0.75 to 1.22 *P* = 1.0000
I‐III Allergen versus CpG [%]	*d* = 0.21 CI:−0.73 to 1.13 *P* = 1.0000	*d* = 0.01 CI:−0.92 to 0.94 *P* = 0.9654	*d* = 0.31 CI:−0.66 to 1.25 *P* = 0.3577

	Nasal discharge	Mucus	Viscosity
I‐II Allergen versus CpG [%]	*d* = 0.28 CI:−0.64 to 1.19 *P* = 0.5712	*d* = 0.23 CI:−0.66 to 1.11 *P* = 0.7033	*d* = 0.04 CI:−0.84 to 0.92 *P* = 0.9696
I‐III Allergen versus CpG [%]	*d* = 0.34 CI:−0.61 to 1.26 *P* = 0.3782	*d* = 0.61 CI:−0.32 to 1.48 *P* = 0.2322	*d* = 0.68 CI:−0.25 to 1.55 *P* = 0.1800

### Breathing type

The breathing type in the allergen group was moderately reduced by 39% after seven inhalations (Fig. 2, Tables 2 and 3). This effect increased in the subsequent six weeks to a 51% improvement in comparison to the initial value (Fig. 2, Tables 2 and 3). The effect was comparable in the CpG only group, with an improvement of 32%, and corresponded to the large clinical reduction of symptoms of 48% in comparison to the initial value (Fig. 2, Tables 2 and 3).

### Lung auscultation

In the allergen group, the pulmonary auscultation revealed a mild improvement of 23% after seven inhalations (Fig. 2, Tables 2 and 3). This effect doubled (Fig. 2, Tables 2 and 3) to 46% improvement over the subsequent 6 weeks in comparison to the first examination. In the CpG only group, a similar improvement (22%) in the clinical findings occurred after the inhalation treatment (Fig. 2, Tables 2 and 3). The clinical effect doubled (46%) in the subsequent interval without treatment (Table 2).

### Interpleural pressure

In the allergen group, a very low (6%) improvement of the interpleural pressures was observed (Fig. 2, Tables 2 and 3). This value persisted over 6 weeks (Fig. 2, Tables 2 and 3). The CpG only group showed a mild decrease of 8% in interpleural pressure directly after the inhalation treatment, although a high individual fluctuation was present. Six weeks after treatment, there was no further change (Fig. 2, Tables 2 and 3).

### Oxygen partial pressure (PaO_2_)

In the allergen group, a mild increase (6%) in oxygen partial pressure in arterial blood was determined after seven inhalations (Fig. 2, Tables 2 and 3). This effect doubled (12%) in the subsequent 6 weeks. The CpG only group showed an initial low increase (4%), which tripled in the subsequent 6 weeks to a value of 12% (Fig. 2, Tables 2 and 3).

### Alveolar‐arterial gradient (AaDO_2_)

The allergen group showed low decrease of the AaDO_2_ of 15% after seven inhalations (Fig. 2, Tables 2 and 3). After 6 weeks a large improvement (45%) from the initial examination was detected (Fig. 2, Tables 2 and 3). In the CpG only group, no improvement was observed directly after the inhalation treatment (Fig. 2, Tables 2 and 3). A moderate improvement (29%) was only observed after the subsequent 6 weeks without further treatment (Fig. 2, Tables 2 and 3).

### Nasal discharge

The nasal discharge decreased with a large clinical effect after seven inhalations by 52% (Fig. 2, Tables 2 and 3) in the allergen group, and almost persisted (45%) over the subsequent 6 weeks. In the CpG only group, this decrease was moderate with 38% (Fig. 2, Tables 2 and 3). This effect also almost persisted (31%) over 6 weeks (Tables 2 and 3).

### Quantification of secretion (Mucus)

In the allergen group, a large clinical decrease by 42% in the quantity of secretion in the trachea was observed after seven inhalations (Fig. 2, Tables 2 and 3). This effect persisted and improved over 6 weeks with a 60% decrease in comparison to the initial value. In the CpG only group, a moderate decrease in quantity of secretion was also observed, with a decrease of 31% after seven inhalations with a persistent effect (35%) demonstrable over 6 weeks (Fig. 2, Tables 2 and 3).

### Viscosity of secretion

In the allergen group, a large clinical decrease of 41% in the viscosity of secretion was observed after the seventh inhalation treatment (Fig. 2, Tables 2 and 3). This continued to improve in the subsequent six weeks without further treatment to 60% in comparison to the initial value (Fig. 2, Tables 2 and 3). A large clinical improvement was also observed in the CpG only group (40%) after seven inhalations with a persistent effect demonstrable over 6 weeks (Fig. 2, Tables 2 and 3).

### Percentage of neutrophils

The allergen group showed a mild decrease in the percentage of neutrophilic granulocytes in the respiratory tract after seven inhalations by 10% (Fig. 2, Tables 2 and 3). This effect improved slightly in the 6 weeks without treatment to 17% reduction. The CpG only group showed no improvement of neutrophils (Fig. 2, Tables 2 and 3) with high individual scattering. After 6 weeks, a very mild increase of the neutrophilic granulocytes in the respiratory tract was determined in comparison to the initial value (Tables 2 and 3).

### Horses summarized

**Figure 3:**
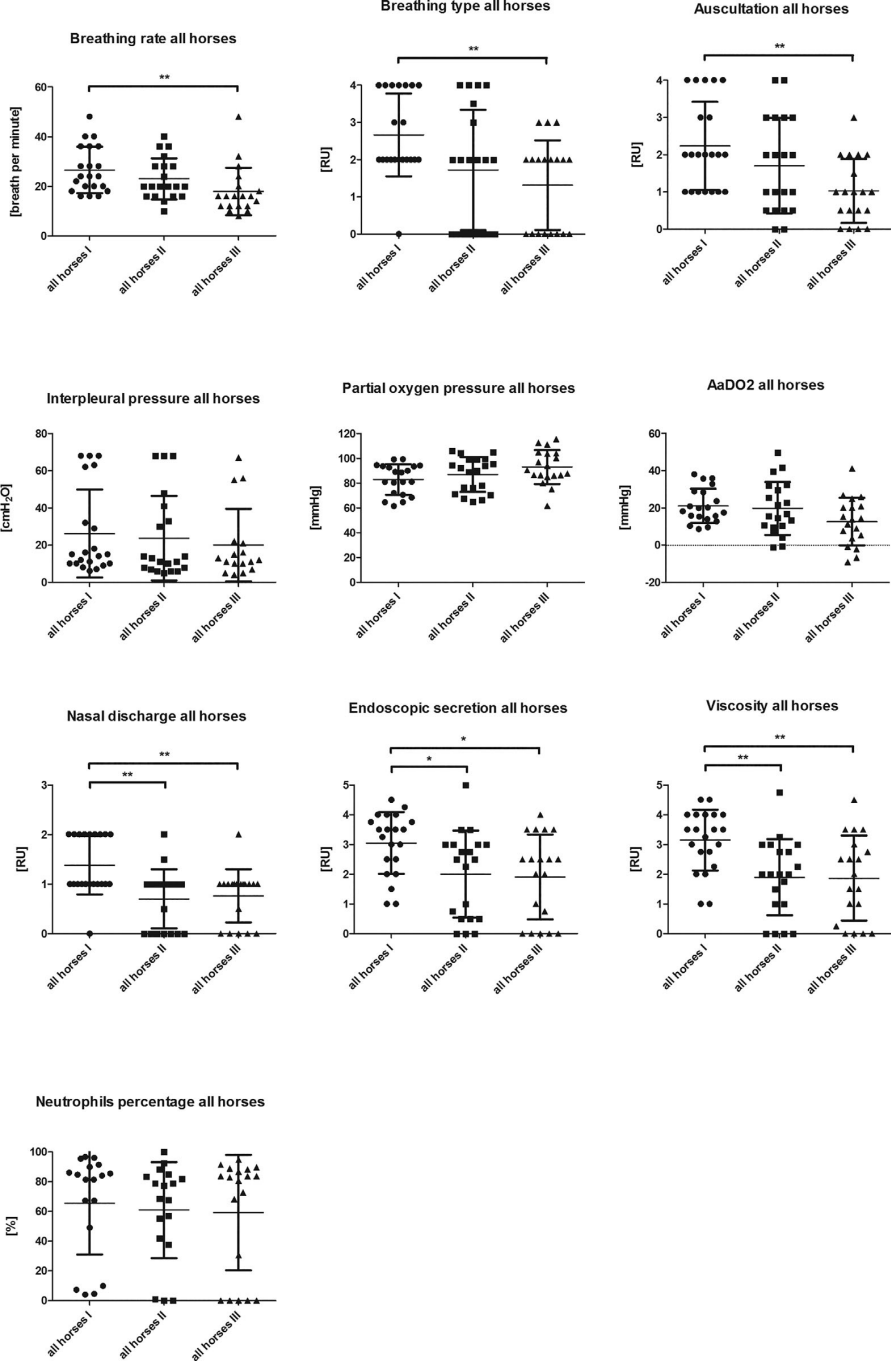
Absolut values of all CpG‐GNP treated horses (*n* = 20) at first (I) examination before inhalation treatment, after seven inhalations (II) and after six weeks without any treatment for evaluation of ongoing effect (III) of breathing rate [1/min], breathing type [RU 0–4], lung auscultation [RU 0–4], interpleural pressure measurement [cmH2O], oxygen partial pressure (PO2) [mmHg], AaDO2 [mmHg], neutrophil percentage of TBS [%], nasal discharge [RU 0–3], endoscopic quantification of secretion (mucus) [RU 0–5] and viscosity [RU 0–5] (calculated value of *P* < 0.05 displayed as *; *P* < 0.01 as **; *P* < 0.001 as ***; mean ±SD).

If all CpG‐GNP treated horses are summarized, independent of additional allergen administration (because of no statistical significant difference between the groups) high significant improvements directly after inhalation treatment and after 6 weeks without further treatment could be detected concerning breathing rate, breathing type, partial oxygen pressure, nasal discharge, auscultation, amount of mucus, and viscosity within the trachea (Fig. 3, Table 4).

**Table 4:** Cohen‘s d, confidence interval (CI) and *P*‐value from all horses calculated from breathing rate, breathing type, lung auscultation, interpleural pressure measurement, oxygen partial pressure (PO2), AaDO2, neutrophil percentage of TBS, nasal discharge, endoscopic quantification of secretion (mucus) and viscosity from absolute values between first and second (I–II) or first and third (I–III) examination from all CpG treated horses (allergen group and CpG only group) summarized (calculated value of *P* < 0.05 displayed as *; *P* < 0.01 as **; *P* < 0.001 as ***)

	Breathing rate	Breathing type	Auscultation	Interpleural pressure
I‐II All horses	*d* = 0.41 CI:−0.22 to 1.02 *P* = 0.3918	*d* = 0.73 CI:0.09–1.35 *P* = 0.0096**	*d* = 0.4 CI:−0.23 to 1.01 *P* = 0.0929	*d* = 0.10 CI:−0.51 to 0.71 *P* = 0.2303
I‐III All horses	*d* = 0.92 CI:0.25–1.55 *P* = 0.0146*	*d* = 1.22 CI:0.52–1.87 *P* = 0.0015**	*d* = 1.12 CI:0.43 to 1.77 *P* = 0.0030**	*d* = 0.28 CI:−0.37 to 0.92 *P* = 0.0712

	AaDO_2_	PO_2_	Neutrophil percentage
I‐II All horses	*d* = 0.12 CI:−0.51 to 0.73 *P* = 0.2862	*d* = 0.30 CI:0.33–0.92 *P* = 0.0671	*d* = 0.14 CI:−0.52 to 0.79 *P* = 0.1556
I‐III All horses	*d* = 0.76 CI:0.09 to 1.39 *P* = 0.0147*	*d* = 0.77 CI:0.10–1.40 *P* = 0.0079**	*d* = 0.18 CI:−0.47 to 0.82 *P* = 0.4777

	Nasal discharge	Mucus	Viscosity
I‐II All horses	*d* = 1.17 CI:0.48–1.8 *P* = 0.0036**	*d* = 0.79 CI:0.14–1.41 *P* = 0.0019**	*d* = 1.04 CI:0.37–1.67 *P* = 0.0011**
I‐III All horses	*d* = 1.08 CI:0.40–1.72 *P* = 0.0073**	*d* = 0.91 CI:0.24–1.54 *P* = 0.0006***	*d* = 0.99 CI:0.32–1.63 *P* = 0.0014**

## Cytokines

### Interleukin‐4

**Figure 4:**
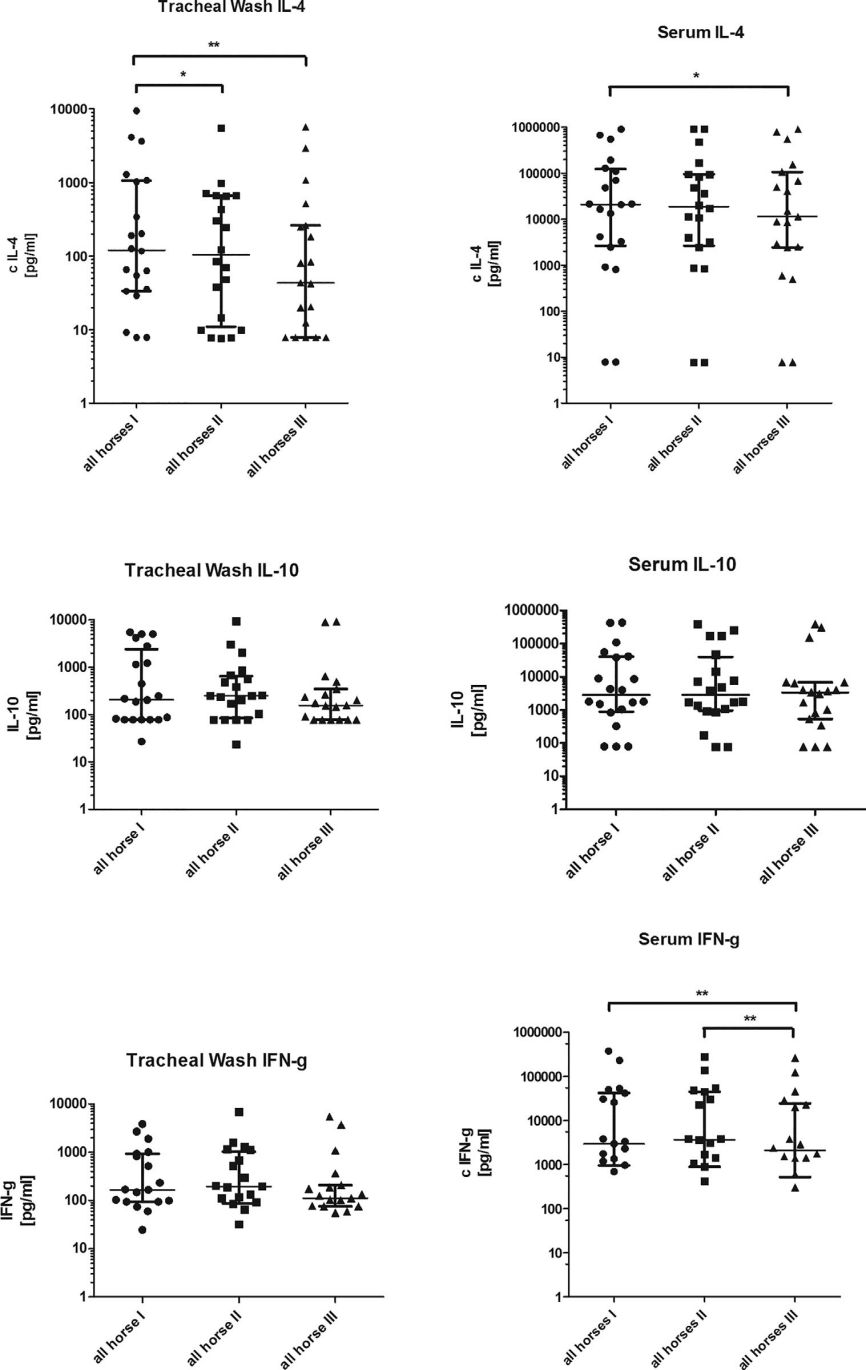
IL‐4, IL‐10, and IFN‐g concentration [pg/ml] in tracheal wash samples and serum of equine asthma‐affected horses at first (I) examination before inhalation treatment, after seven inhalations (II) and after 6 weeks without any treatment to evaluate ongoing effect (III). All horses (*n* = 20) independent of the group are depicted. Serum IFN‐g all horses: nine points are under detection limit. Tracheal wash IFN‐g: five points are under detection limit. Calculated value of *P* < 0.05 displayed as *; *P* < 0.01 as **; *P* < 0.001 as *** (individual values as scattered dot plot and median with interquartile range are depicted).

The concentration of the cytokine IL‐4 varied greatly among the horses from 7.8 pg/ml to 900,000 pg/ml, and thus was partially below and above the limit of detection (15.6–2,000 pg/ml). Overall, IL‐4 was downregulated (Fig. 4). The concentration of IL‐4 in the tracheal wash sample was reduced after the last inhalation, independent of the group (*P* = 0.0217) and also decreased after another 6 weeks (*P* = 0.0070), in comparison to the initial value. In comparison to the initial value, the median decrease was 14% directly after the inhalation treatment, and 64% 6 weeks after the last inhalation. In the serum, a decrease in the concentration of IL‐4 could only be observed after 6 weeks (a median decrease of 45%, *P* = 0.0186). Therefore, a long‐term effect over 6 weeks could be observed both in the serum as well as in the tracheal wash sample, independent of the group (Fig. 4).

### Interleukin‐10

The concentrations of IL‐10 greatly varied initially among the horses from 78 pg/ml to >400,000 pg/ml, and were in part above or below the limit of detection (156.2–20,000 pg/ml). There was a short upregulation of IL‐10 in the tracheal sample directly after treatment. In contrast, the IL‐10 upregulation in the serum sample was delayed. Over the 6 week period the concentration of IL‐10 decreased in the tracheal wash sample comparable to the decrease of IFN‐γ. In the local tracheal wash sample there was a median increase of IL‐10 of 21% after inhalation treatment in comparison to the baseline. After 6 weeks a decrease of 37% could be detected in comparison to the sample taken after inhalation treatment. Altogether IL‐10 showed a decrease of 25% from the first to the last examination. In the serum samples, independent of the groups, a median increase of 20% could only be detected 6 weeks after inhalation treatment in comparison to the baseline (Fig. 4). However, none of those changes were statistical significant.

### Interferon‐γ

A median increase of 16% in concentration of the Th1‐type cytokine IFN‐γ could be detected directly after the inhalation therapy in the tracheal wash sample. In contrast, a decrease of 43% was observed after 6 weeks in comparison to the sample taken directly after treatment (Fig. 4). The median of the relative decrease was 33% in comparison to the baseline. However, none of those changes were statistically significant.

In the serum sample, a nonsignificant median increase of 23% could be detected directly after the inhalation treatment. After 6 weeks there was a median decrease of 43% in comparison to the sample taken directly after inhalation treatment (*P* = 0.0057). Altogether, a demonstrable decrease in IFN‐γ of 30% could be seen after 6 weeks in comparison to the baseline (Fig. 4) (*P* = 0.0035).

### Interleukin‐17

The concentration of IL‐17 was only measured in the local tracheal wash samples. The values measured diverged greatly within and between both groups, in the initial concentrations, as well as in the samples taken directly after the inhalation treatment and 6 weeks later, with no significant changes between time points. In addition, there was no significant change of the IL‐17 concentrations and the neutrophilic granulocytes in the tracheal wash samples.

### Comparison between the groups

Comparing the two groups, there was no significant difference in the concentrations of cytokines at any time point.

## Discussion

Nanoparticle‐bound CpG‐ODN immunotherapy offers an allergen‐independent effective and innovative treatment approach of equine asthma‐affected horses with ongoing clinical improvement.

According to the results of this study there seemed to be no need for allergy testing and adding allergens to the CpG‐GNP immunotherapy. In this study it was possible to achieve the same clinical and immunological effects with exclusive CpG‐GNP therapy. This is particularly relevant in light of the fact that it is tremendously difficult to determine the clinical relevance of the positive test reactions for individual allergens.

The combination of specific allergens and the immunomodulatory CpG‐ODN is similar to what was reported by Senti et al. 6 in human patients with allergic asthma and rhinitis. In that study, CpG‐ODN was packed in virus‐like particles as adjuvants, and administered subcutaneously with house dust mite allergens, which led to a clinical remission of at least 38 weeks 6.

The use of nanoparticle‐bound CpG‐ODN for immunomodulatory monotherapy of horses with equine asthma has already been reported 6, 7. The biodegradable gelatin nanoparticles are well tolerated, immunologically inert, stable for inhalation, and a successful drug delivery system 7, 8, 20.

In the present study, a functional in‐vitro test (FIT) was conducted to assist in the identification of offending allergens, in addition to history and physical examination. The results of this test varied greatly which corresponds to the results of other studies 21, 22, 23, 24, 25, 26, 27, 28. Numerous horses reacted to pollen antigens, which should be associated with seasonal deterioration in the spring and summer months. However, the clinical signs occurred predominantly year‐round.

As determined by Bruennlein 26, the most frequent positive reactions of the equine asthma horses examined occur upon exposure to dust and storage mites. In particular, mold spores were shown to play an important role in the allergic reactions of equine asthma horses 22, 23, 24, 25, 28, 29, 30. Three horses showed no reactions in the FIT. Possibly, their offending allergens were not represented in the limited selection of tested allergens. Alternatively, there was no allergic type I hypersensitivity component to their disease which is the only type of hypersensitivity that commercially available assays test for. Most likely types III and IV immune reactions are also involved in the pathogenesis of equine asthma 31 and those are missed with tests directly or indirectly measuring IgE. IgE‐mediated type I hypersensitivity reactions are acute immune reactions with mast cell degranulation due to cross‐linking of cell‐bound IgE antibodies by antigens. This is in contrast to immune complex (antibody‐antigen complex) type III reactions with an inflammatory response in blood vessels and tissue characterized by complement fixation and ligation of Fc receptors on leucocytes. Finally, type IV reactions are cell‐mediated, delayed reactions mediated by T cells. Furthermore, allergy tests prove only sensitization to an allergen, but not its clinical relevance 26. This can only be demonstrated by a provocation test 32, which in most cases is not possible for practical and ethical reasons.

An excessive pro‐allergic Th2 immune response cannot be documented in all cases of equine asthma 33, probably due to individual factors such as exacerbation phase, duration of the disease, genetic, and exogenous trigger factors and allergen load 34. The initial concentration of IL‐4 in the horses varied greatly. This is in accordance with the results of others studies 35, 36, 37, 38, 39 and shows the heterogeneous immunological phenotypes of the disease.

A downregulation of the Th2 immune response and thus the IL‐4 secretion is expected by CpG‐ODNs through the activity of TLR9 6, 7, 40, 41, 42, 43. Since IL‐4 causes class switching and IgE production in B cells 31, 44, 45, 46, its downregulation is an important goal of the CpG‐ODN immunotherapy. However, the involvement of IgE in the pathogenesis of equine asthma could not be shown in all studies 31, it probably is only one aspect of this multifactorial disease. Furthermore, type I, III, and IV immune responses are probably involved in equine asthma, depending on the phase of disease and individual variations 31, 47.

A recent pilot study showed clinical improvement of horses with insect bite hypersensitivity after pure allergen treatment 48. Studies in which CpG‐ODN was combined with an allergen for immunotherapy showed that the IL‐4 concentration decreased 44, 49, or interferon‐gamma increased 50. This is in accordance with our study, where a decrease of IL‐4 in tracheal washes could be documented directly and 6 weeks after the end of treatment. The IL‐4 serum concentrations were markedly decreased only in the final examination 6 weeks post treatment. Adding allergens to the CpG‐ODN inhalation showed no additional effect. This corresponds to findings of other groups 52. Higher allergen concentrations or longer duration of the hyposensitization may have led to different results 6, 53.

Overall, it may be concluded that a long‐term decrease of the pro‐allergic Th2 cytokine IL‐4 is a promising result of an immunomodulatory inhalation treatment for equine asthma.

CpG‐ODN is able to activate specific Treg cells and increase the release of IL‐10 54, which can inhibit the Th2 as well as the Th1 immune response and thus the clinical signs 10, 55. Allergen immunotherapy has been shown to increase IL‐10 in cats with asthma 43 and dogs with atopic dermatitis 56. IL‐10 only showed a short increase in the tracheal wash sample, possibly due to an insufficient IL‐10 response and a deficiency of Treg cells, similar to what is reported for human asthma 57. In contrast, IL‐10 demonstrated a lasting increase in the serum sample. In a previous in‐vitro study, BAL cells of healthy horses formed more IL‐10 after CpG‐ODN stimulation than those of equine asthma horses 12. In contrast, an in‐vivo study showed a significant increase of IL‐10 in the BAL of equine asthma horses after five inhalations of CpG‐ODN 7. Regardless of the IL‐10 concentrations, inhalation of CpG‐ODN can achieve a persistent improvement of clinical signs in horses with equine asthma as shown in this study and former studies 7, 8.

In this study, IFN‐γ concentration in the tracheal wash or in the serum showed a short increase directly after inhalation treatment followed by a decrease in both groups after 6 weeks. This contrasts with a previous study 7 and the expected Th2/Th1 shift 40, but indicates a reduction of the inflammatory response with a long‐term effect over 6 weeks 10, 18, 40, 58.

IL‐17 is a chemotactic and activating molecule for neutrophils, which are crucially involved in the pathogenesis of equine asthma. It also stimulates the mucus secretion in the bronchi 59, 60, 61. In this study, it did not decrease in the tracheal wash samples. There was also no direct correlation to the percentage of neutrophils in the TBS. Furthermore, the initial tracheal concentrations of IL‐17 varied greatly among horses. This fits to the reported local variations of cytokines in asthma‐afflicted horses probably due to genetic factors, the disease stage, and varying environmental allergen exposures 35, 36, 37, 38, 39, 62.

How much the immunological effects of the inhalation with CpG‐GNP in this study were achieved by the treatment and how much seasonal weather changes such as colder temperatures and damp weather with a decreased environmental allergen load were contributing to the changes is unclear 2, 51. This could have been excluded by the continuous stabling of the horses during the study, but would not have corresponded with the practical objectives of the study.

In this study protocol the number of inhalations was increased from five to seven, compared to a previous study 8, to examine the influence of more inhalations on dyscrinia and hypercrinia, percentage of neutrophils in the tracheal wash, arterial blood gas values and duration of the clinical improvement. Similarly, the follow‐up examinations were extended from 4 8 to 6 weeks post treatment, as owners from the previous study had reported a longer persistence of their horses’ clinical improvement than 4 weeks, which could be confirmed in this study. In addition, the increase from five 8 to seven inhalations demonstrated a further improvement in clinical parameters, a decreased percentage of neutrophils in the trachea, amount of secretion and viscosity, as well as an increased partial oxygen pressure in arterial blood gas compared to the former study 8.

The gradual increase in the allergen dose was conducted similar to what is recommended for subcutaneous allergen immunotherapy for atopic dermatitis 63 and was determined in another experiment (data not shown) with the aid of a histamine inhalation provocation test to avoid any bronchospasm or signs of dyspnea with the inhaled allergens. The allergen dosage used was relatively low. In combination with the low number of total applications this was very different from a classical immunotherapy, which extends over a period of months. The classical subcutaneous route of administration would of course be an alternative and avoid exposing the lungs directly to the allergen concentration.

Horses were left in their usual stabling environment during this field study. A homogeneous group of patients with identical duration of disease, individual triggers, and sensitivities and environmental exposures is difficult to obtain for this multifactorial disease. In addition, this field study evaluated a clinical response of a treatment in practice and thus should reflect the conditions seen in this environment. As a consequence limitations of the study are the heterogeneous horse population (age, breed, duration of disease, different barns, and different bedding materials), the relatively small number of horses and the exploratory character of the field study. Overall, treatment of equine asthma in horses offers many challenges such as identifying the clinically relevant allergens from allergy tests and addresses the multifactorial pathogenesis and the manifold exogenous environmental factors influencing the disease. However, CpG‐GNP, with or without allergens, provides an inhalation immunotherapy that seems effective over at least six weeks for treatment‐resistant horses with chronic respiratory disease and may be of interest for the therapy of human asthma.

## Acknowledgement

The authors thank Ms. Elena Serkin for critical revision of the paper, and Prof. Christian Plank for kindly providing lab facilities for conducting ELISAs at the Institute of Experimental Oncology and Therapy Research at Klinikum Rechts der Isar, TU Munich. The authors further thank Inspiration Medical for kindly providing the Aeroneb® Go micropump nebulizer and Artu Biologicals Europe B.V. for providing the allergens. Parts of the research were supported by the Deutsche Forschungsgemeinschaft (DFG) (Germany) (GE’2044/4‐1). The Aeroneb® Go micropump nebulizer (Aerogen, Galway, Ireland) was kindly sponsored by Inspiration Medical (Bochum, Germany). The allergens were kindly sponsored by Artu Biologicals Europe B.V. (Lelystad, the Netherlands).

## Authors’ Contribution

Authors (Klier, Geis, Steuer) contributed equally. The study was conducted at the Equine Clinic, Centre for Clinical Veterinary Medicine, LMU Munich, Germany. The ELISAs were performed by JS in the lab at the Institute of Experimental Oncology and Therapy Research, Klinikum Rechts der Isar, Technical University, Munich. Abstracts of this study were presented in part at the 2015 Deutsche Gesellschaft für Pneumologie und Beatmungslehre (DGP) and Deutsche Veterinärmedizinische Gesellschaft (DVG) congress, Berlin, Germany; the 2015 British Equine Veterinary Association (BEVA) congress, Liverpool, England; the 8th Leipziger Tierärztekongress 2016, Leipzig, Germany; and the Inn Lab Tagung 2016 of the Deutsche Veterinärmedizinische Gesellschaft (DVG), Berlin, Germany.

## Conflicts of Interest

The authors declare no competing financial interests.

## Owners’ Agreement

All horse owners signed a consent form.

## Off‐Label Antimicrobial Declaration

Authors declare no off‐label use of antimicrobials.

## Notes

1 Biomers GmbH, Ulm, Germany.

2 Equine HealthCare Aps, Hoersholm, Denmark.

3 Aerogen, Galway, Ireland.

4 Diametrics Medical incorporated, Keller Medical, Bad Soden, Germany.

5 Medion diagnostics, Düdingen, Switzerland.

6 Boehringer Ingelheim, Germany.

7 R&D systems, Minneapolis, USA.

8
http://davidmlane.com/hyperstat/effect_size.htm, Robert Coe.

9 GraphPad software Inc., La Jolla.
